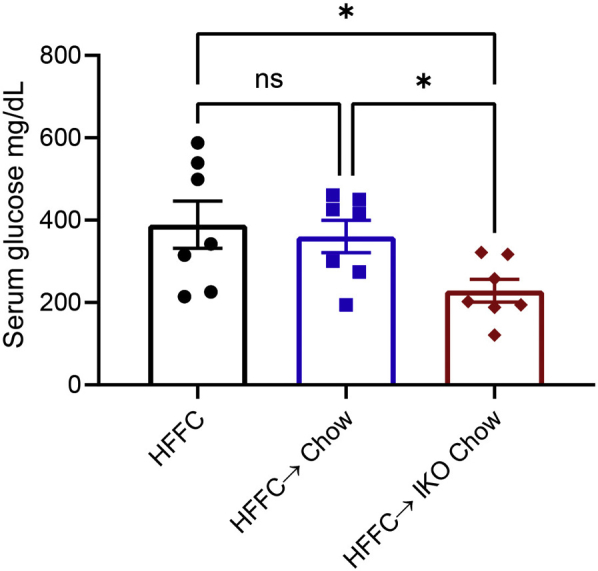# Inhibition of chylomicron assembly leads to dissociation of hepatic steatosis from inflammation and fibrosis

**DOI:** 10.1016/j.jlr.2021.100123

**Published:** 2021-09-24

**Authors:** Yan Xie, Elizabeth P. Newberry, Elizabeth M. Brunt, Samuel J. Ballentine, Saeed Soleymanjahi, Elizabeth A. Molitor, Nicholas O. Davidson

**Affiliations:** 1 Division of Gastroenterology, Department of Medicine, Washington University School of Medicine, St. Louis, MO, USA; 2 Department of Pathology and Immunology, Washington University School of Medicine, St. Louis, MO, USA

**Keywords:** intestine, lipid absorption, antioxidant, apolipoprotein B, lipoproteins, microsomal triglyceride transfer protein, fibrosis, oxidative stress, intestinal permeability, hepatic lipogenesis, ALT, alanine aminotransferase, Cer, ceramide, DNL, de novo lipogenesis, HFFC, high-fat, high-fructose, high-cholesterol diet, JNK, c-Jun N-terminal kinase, MCD, methionine/choline-deficient diet, *Mttp-IKO*, microsomal triglyceride transfer protein knockout mice, NAFLD, nonalcoholic fatty liver disease, NASH, nonalcoholic steatohepatitis, NF-κB, nuclear factor kappa B, QC, quality control, SCD, steroyl CoA desaturase, TAM, tamoxifen, TBARS, thiobarbituric acid reactive substance, TG, triglyceride

## Abstract

Regulating dietary fat absorption may impact progression of nonalcoholic fatty liver disease (NAFLD). Here, we asked if inducible inhibition of chylomicron assembly, as observed in intestine-specific microsomal triglyceride (TG) transfer protein knockout mice (*Mttp-IKO*), could retard NAFLD progression and/or reverse established fibrosis in two dietary models. *Mttp-IKO* mice fed a methionine/choline-deficient (MCD) diet exhibited reduced hepatic TGs, inflammation, and fibrosis, associated with reduced oxidative stress and downstream activation of c-Jun N-terminal kinase and nuclear factor kappa B signaling pathways. However, when *Mttp*^*flox*^ mice were fed an MCD for 5 weeks and then administered tamoxifen to induce *Mttp-IKO*, hepatic TG was reduced, but inflammation and fibrosis were increased after 10 days of reversal along with adaptive changes in hepatic lipogenic mRNAs. Extending the reversal time, following 5 weeks of MCD feeding to 30 days led to sustained reductions in hepatic TG, but neither inflammation nor fibrosis was decreased, and both intestinal permeability and hepatic lipogenesis were increased. In a second model, similar reductions in hepatic TG were observed when mice were fed a high-fat/high-fructose/high-cholesterol (HFFC) diet for 10 weeks, then switched to chow ± tamoxifen (HFFC → chow) or (HFFC → *Mttp-IKO* chow), but again neither inflammation nor fibrosis was affected. In conclusion, we found that blocking chylomicron assembly attenuates MCD-induced NAFLD progression by reducing steatosis, oxidative stress, and inflammation. In contrast, blocking chylomicron assembly in the setting of established hepatic steatosis and fibrosis caused increased intestinal permeability and compensatory shifts in hepatic lipogenesis that mitigate resolution of inflammation and fibrogenic signaling despite 50–90-fold reductions in hepatic TG.

Nonalcoholic fatty liver disease (NAFLD) is the most common form of chronic liver disease worldwide, affecting more than a quarter of the US population (1). A subset of patients with NAFLD will progress to develop nonalcoholic steatohepatitis (NASH) with inflammation and fibrosis, and a further subset will develop cirrhosis and hepatocellular cancer, through mechanisms and pathways that have yet to be fully understood (2). The pathogenesis of NAFLD and its progression to NASH involves genetic and environmental interactions (3) as well as signaling events between and among cells in the liver (hepatocytes, stellate cells, and resident and circulating immune cells) and with other organs such as adipose tissue and intestine (2). Altered homeostatic signaling across the gut-liver axis affects hepatic lipid metabolism as well as immune signaling and inflammation, through mechanisms including (but not limited to) altered fat absorption, changes in gut microbiota, disrupted intestinal permeability, incretin, and bile acid signaling (4, 5). Those findings have promoted interest in strategies for prevention and resolution of NASH by targeting the gut-liver axis, with findings in preclinical models and human subjects showing attenuated hepatic steatosis but no consistent or sustained effects on hepatic inflammation and fibrosis (6, 7, 8). Studies in preclinical models support a role for alterations in gut-liver signaling in the progression of NASH, including work showing that mice fed high-fat diets and exposed to dextran sodium sulfate develop increased gut bacterial translocation and worsened hepatic fibrosis (9). Other work has highlighted a role for inflammasome signaling associated with altered gut microbial taxa in promoting hepatic steatosis and inflammation (10).

In regard to the impact of impaired intestinal lipid transport on NAFLD, we and others have shown that mice with conditional intestinal deletion of microsomal triglyceride (TG) transfer protein (*Mttp-IKO*) demonstrate impaired chylomicron assembly and decreased intestinal fat (TG and cholesterol) absorption with decreased hepatic steatosis, despite increased hepatic de novo lipogenesis (DNL) and increased VLDL secretion (11, 12, 13). In addition, conditional *Mttp-IKO* mice are protected from developing fructose-induced hepatic steatosis through multiple adaptations including increased incretin secretion, altered microbial taxa, and shifts in bile acid signaling (14). However, the extent to which inducible impairment of chylomicron assembly and altered gut-liver signaling in *Mttp-IKO* mice mitigates either the development or the reversal of hepatic inflammation and fibrosis is unknown. Other findings in the setting of impaired chylomicron assembly and secretion suggest that intestinal *Mttp* deletion impairs immune signaling in both mice and humans (15, 16). In addition, yet other findings suggest that *Mttp-IKO* mice exhibit a greater intestinal tumor burden in colitis-associated cancer (17). Those findings collectively point to a series of adaptations in immune signaling and inflammation in *Mttp-IKO* mice that could conceivably exaggerate fibrogenic signaling and exacerbate liver injury in mice, even in the absence of established steatosis and fibrosis.

Here, we asked if inducible deletion of chylomicron assembly in *Mttp-IKO* mice could mitigate NAFLD/NASH progression and/or reverse established fibrosis, using an approach incorporating two distinct dietary models. In the first model, we fed mice a methionine/choline-deficient (MCD) diet as a model of inflammatory and fibrogenic injury (18). MCD feeding induces a robust fibrogenic response but does not replicate other features of NAFLD including obesity and insulin resistance (18). In the second model, we fed mice a high-fat, high-fructose, high-cholesterol (HFFC) diet, which is a nutritional model of obesity-associated steatosis and fibrogenic injury (19, 20). Our findings show that blocking lipid absorption attenuates MCD-induced NAFLD progression by reducing steatosis, oxidative stress, and inflammation. However, while blocking lipid absorption decreases established steatosis in both dietary models, with over a >90-fold range of hepatic TG content, fibrosis, and inflammation were unchanged. Our findings suggest that increased intestinal permeability and compensatory shifts in hepatic lipogenesis likely combine to promote continued inflammation and fibrogenic signaling despite the reduction in hepatic steatosis.

## Materials and methods

### Animals and diets

All animal protocols were approved by the Washington University IACUC and conformed to standards outlined in the Guide for the Care and Use of Laboratory Animals (the National Institutes of Health, 1985) and conducted in accordance with institutional guidelines (IACUC #20180284). Mice were housed in ventilated cages on a 12 h light/dark cycle with corncob bedding and ad libitum access to rodent chow (PicoLab Rodent Diet 20; LabDiet, St Louis, MO) and water unless otherwise noted. *Mttp*^*f/f*^ Villin Cre ER^T2^ mice (11) were injected with tamoxifen (TAM) at 50 μg/g body weight/day/5 days with littermates receiving vehicle injection as control. Both male and female mice were used, with the majority of experiments conducted in male mice as indicated in the relevant legends to the figures (11). In a first dietary model, 12–16-week-old *Mttp*^*f/f*^ Villin Cre ER^T2^ mice were fed MCD (MP Biomedicals, LLC; catalog no.: 960439) for 3 weeks, starting 3 weeks after TAM or vehicle injection. Where indicated, other groups of *Mttp*^*f/f*^ Villin Cre ER^T2^ mice were fed MCD for 5 weeks with groups of mice subsequently injected with TAM or vehicle and sacrificed either 10 days later (while consuming MCD) or switched to regular rodent chow and sacrificed 35 days later. In a second dietary model, *Mttp*^*f/f*^ Villin Cre ER^T2^ mice were fed an HFFC diet (Research Diets, Inc; catalog no.: D09100301) for 10 weeks, then switched to regular rodent chow and injected with either TAM or vehicle, and sacrificed 35 days later. At sacrifice, blood and tissues were collected after 4–6 h fasting and frozen at −80^o^C. Portions of freshly isolated tissue were taken for processing as described later.

### Histologic analysis

Livers were fixed in 10% neutral-buffered formalin, sectioned, and stained with H&E or used for Sirius red or anti-F4/80 antibody immunohistochemical staining (1:100 dilution) as previously detailed (20, 21) using Nuance 2.10 multispectral imaging (PerkinElmer) or ImageJ software (the National Institutes of Health).

### Hepatic lipid, collagen content, and oxidative stress parameters

Hepatic TG, cholesterol, and FFAs were determined enzymatically using commercial kits (FUJIFILM Wako Diagnostics, Richmond, VA) as previously described (20, 21). Hepatic collagen content was determined using a commercial kit (QuickZyme Biosciences; catalog no.: QZBTOTCOL1), following instructions with minor modifications. In brief, about 80 mg of snap-frozen liver was homogenized in 1 ml water, and an equal volume of 12 N HCl added to the homogenate and hydrolyzed at 95^o^C for 20 h. Collagen content in the mixture was measured spectrometrically and normalized to protein content. Hepatic lipid hydroperoxide and glutathione levels were measured on fresh liver samples after homogenization using a commercial kit (catalog Nos.: 705002 and 705003; lipid hydroperoxide assay kit [catalog no.: 703002]; glutathione assay kit [Cayman Chemical, Ann Arbor, MI]). Hepatic thiobarbituric acid reactive substances (TBARS) were determined by Parameter™ TBARS kit (R&D Systems; catalog no.: KGE013) following the manufacturer's instructions with modifications for use on lipid-rich samples. In brief, liver lipids were extracted by chloroform:methanol (2:1). The organic phase was dried under nitrogen and redissolved in 1% Triton. TBARS in the mixture were determined spectrometrically following the manufacturer's instructions.

### RNA extraction, sequencing, and quantitative PCR

For each genotype and condition, three pools were prepared, each containing RNAs from three to four separate mice, for a total of 10 μg RNA per final pool. Pools were sequenced on an Illumina HiSeq system. Base calls and demultiplexing were performed with Illumina's bcl2fastq software and a custom python demultiplexing program with a maximum of one mismatch in the indexing read. RNA-Seq reads were then aligned to the Ensembl release 76 primary assembly with STAR, version 2.5.1a (22). Gene counts were derived from the number of uniquely aligned unambiguous reads by Subread:feature Count, version 1.4.6-p5 (23). Isoform expression of known Ensembl transcripts was estimated with Salmon, version 0.8.2 (24). Sequencing performance was assessed for the total number of aligned reads, total number of uniquely aligned reads, and features detected. The ribosomal fraction, known junction saturation, and read distribution over known gene models were quantified with RSeQC, version 2.6.2 (25). For quantitative PCR determination, RNA was extracted using TRIzol® Reagent (Invitrogen Life Technologies, Carlsbad, CA) and treated with DNase. Genes exhibiting differential regulation were selected and used as input to String-db.org for functional protein association network analysis. Reverse transcription was performed using the ABI high-capacity complementary DNA reverse transcription kit (catalog no.: 4368814), with ∼1 μg of total RNA and random hexamers, to generate complementary DNA. qRT-PCR assays were performed in triplicate on a Step One Plus Sequence Detection System using Fast SYBR Green Master Mix (catalog no.: 4385612; Applied Biosystems). Primer pairs were designed by Primer Express software 3.0 (Applied Biosystems), with sequences listed in supplemental Table S1. Relative mRNA abundance was validated in a subset of targets using qRT-PCR and expressed as fold change compared with mRNA levels in control mice, normalized to GAPDH.

### Intestinal permeability

Mice were administered FITC-labeled dextran (FD-4; molecular weight 4,000; Millipore Sigma; catalog no.: FD4) by oral gavage (400 mg/kg body weight) as described previously (17), with FD-4 levels measured in serum collected 2 and 4 h after gavage using fluorimetry (Synergy HT; BioTek®) at excitation of 485/20 and emission of 528/20. Serum levels of alanine aminotransferase (ALT) and aspartate aminotransferase were measured with kits following manufacturer's instructions (catalog no.: A526-120 and A561-120; TECO DIAGNOSTICS, Anaheim, CA).

### Targeted hepatic lipidomic survey

Liver samples were homogenized in PBS (1:4 w/v) using an Omni bead ruptor (PerkinElmer), and lipid extracted using a modified Bligh-Dyer method (20). Internal standards included ceramide (Cer) (17:0), TG (17:0-17:0-17:0), diglyceride/diacylglycerol (21:0-21:0), phosphatidylcholine (14:1-14:1), and d4-FFA (16:0), respectively. Internal standards were added to all samples before extraction. Three blank samples were prepared and run with the study samples for FFA assay, with the average signal of FFA in blank sample subtracted to eliminate artifactual interference. Quality control (QC) samples were prepared by pooling aliquots of the study samples and used to monitor the instrument stability. The QC was injected three times in the beginning to stabilize the instrument and was injected between every four study samples. Only lipid species with coefficient of variation <15% in QC sample were reported. Measurement of FFA was performed with a Shimadzu 20A HPLC system and a Shimadzu SIL20AC HT autosampler coupled to a 4000QTRAP mass spectrometer operated in positive multiple reaction monitoring mode. Measurement of phosphatidylcholine, diglyceride/diacylglycerol, TG, and Cer was performed with a Shimadzu 20A HPLC system and a Shimadzu SIL-20AC HT autosampler coupled to an API4000 mass spectrometer operated in positive multiple reaction monitoring mode. Data processing was conducted with Analyst 1.6.3 (AB Sciex). The relative quantification of lipids was undertaken with data reported as the peak area ratios of the analytes relative to the corresponding internal standards. DNL index, FA elongation index, and steroyl CoA desaturase (SCD) index were calculated using FA chain length and saturation parameters as previously detailed (20, 26, 27). DNL index is the ratio of FFA 16:0 to 18:2; SCD index is the ratio of (16:1 + 18:1) divided by (16:0 + 18:0); FA elongation index is the ratio of (18:0 + 18:1) divided by (16:0 + 16:1). The comparison of individual lipid species (peak area ratio) was performed using a multiple unpaired *t*-test using GraphPad Prism 9.0.0 (GraphPad Software, Inc).

### Statistical analysis

Data were analyzed using GraphPad Prism 9.0.0 by unpaired *t*-test or Mann-Whitney *U* test. For multigroup comparisons, statistical differences were determined by one-way analysis of variance and Tukey's multiple comparison post hoc test. Pearson correlation coefficients were calculated based on the hepatic TG and fibrotic area by GraphPad Prism 9.0.0. The level of significance was set at *P* < 0.05.

## Results

### *Mttp-IKO* mice fed an MCD diet exhibit reduced hepatic steatosis, inflammation, and fibrosis

**Fig. 1:**
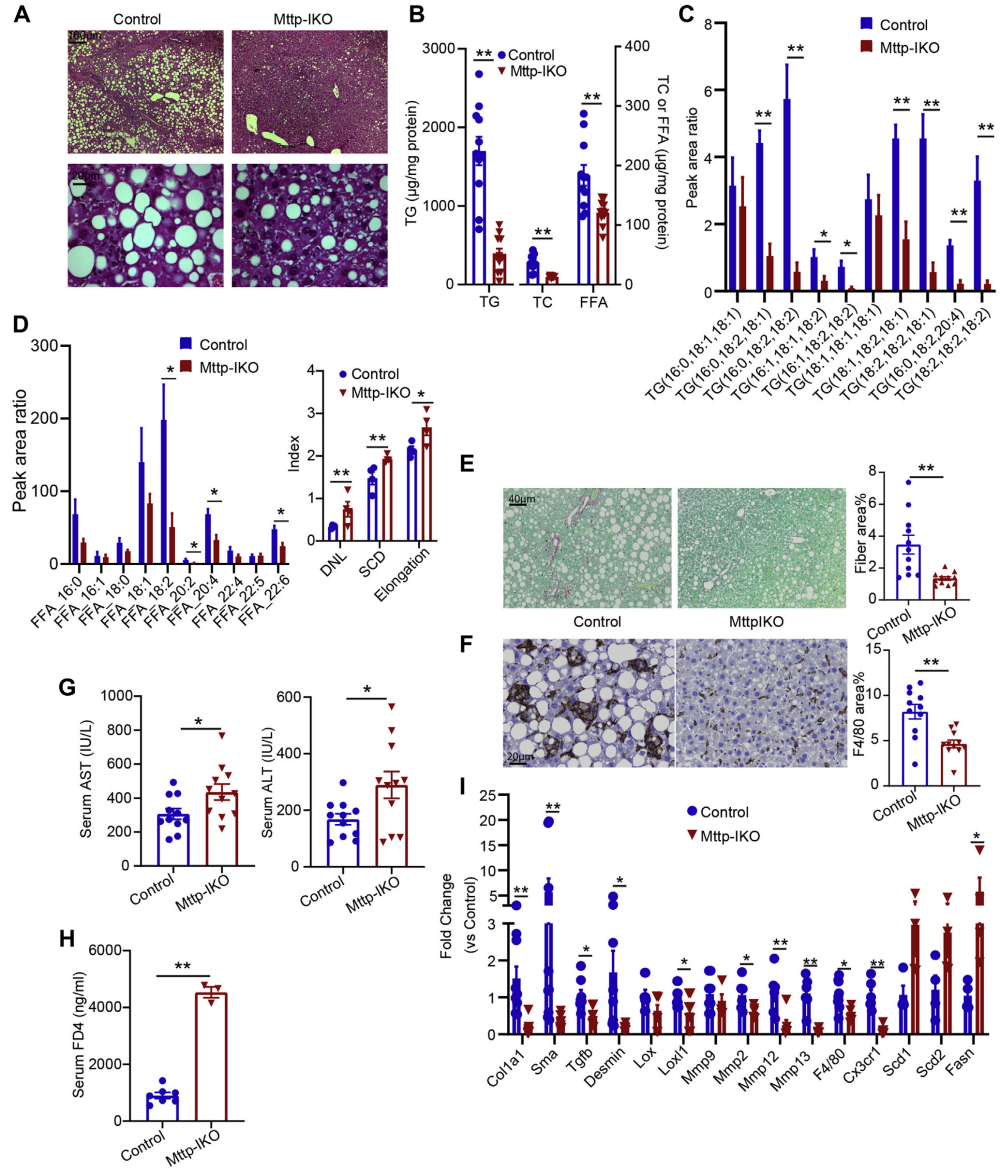
Conditional intestinal *Mttp* deletion prevents hepatic steatosis, fibrosis, and inflammation in male mice-fed MCD for 3 weeks. A: Representative images of H&E-stained liver tissue at 50× (upper panels) and 400× magnification. B: Biochemical quantitation of hepatic lipid content, including TG, FFAs, and total cholesterol (TC) (n = 11/genotype). C: Relative abundance of the most abundant TG species presented as peak area ratio (n = 4/group). D: Relative abundance of the most abundant FFA species presented as peak area ratio (left panel). Right panel shows indices of DNL, FA saturation (SCD), and elongation (n = 4/genotype) calculated from peak area of all FFA species. E: Representative images of Sirius red-stained liver tissue (200× magnification). Right panel shows quantitation of fibrotic area, expressed as percent of total tissue area (n = 11/genotype). F: Representative images of F4/80 staining (400×). Right panel shows quantitation of F4/80-stained area expressed as percent of total tissue area (n = 11/genotype). G: Serum AST (left) and ALT levels (n = 11/genotype). H: Serum FITC-dextran (FD4) levels 2 h after oral gavage (n = 3–7/genotype). I: mRNA expression of genes related to fibrosis, inflammation, and lipogenesis (n = 4–11/genotype). For all panels, data are presented as mean ± SEM, with ∗ indicating *P* < 0.05 and ∗∗ indicating *P* < 0.01. AST, aspartate aminotransferase.

**Supplemental Fig. S1:**
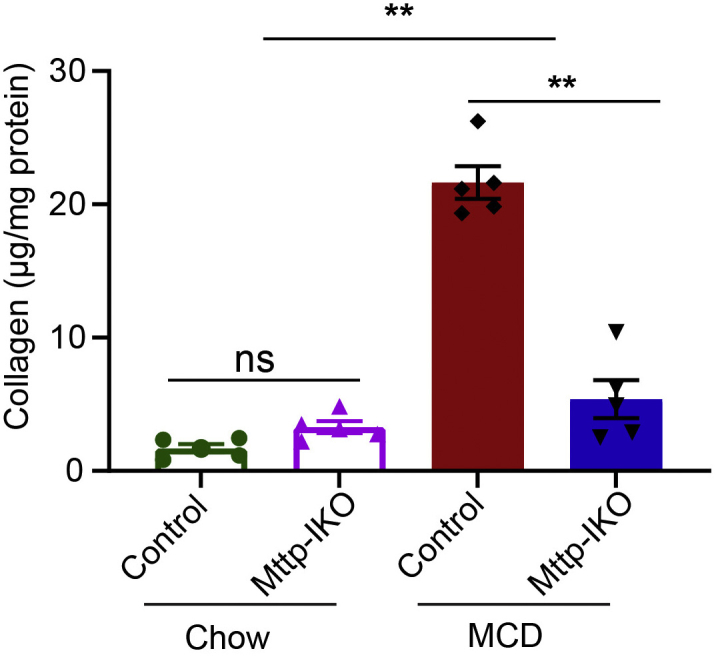


Mice of both genotypes (WT littermate controls and TAM-treated *Mttp*^*f/f*^ Villin Cre ER^T2^ mice) were fed an MCD diet for 3 weeks, revealing macrovesicular steatosis (Fig. 1A) with *Mttp-IKO* mice showing ∼80% reduction (1,700 → 385 μg/mg protein) in hepatic TG content as well as reduced cholesterol and FFA (Fig. 1B). Hepatic FA species distribution was altered in *Mttp-IKO* mice with reduced abundance of polyunsaturated FAs (C18:2, 20:4) evident in both TGs and FFAs (Fig. 1C, D), with a corresponding increase in DNL, SCD, and elongation indices (Fig. 1D). An adaptive increase in hepatic DNL is consistent with our prior findings in chow-fed *Mttp-IKO* mice (11). Hepatic inflammation and fibrosis were also reduced in *Mttp-IKO* mice as evidenced by reduced Sirius red and F4/80 staining (Fig. 1E, F), despite the increase in serum transaminases (Fig. 1G) and increased intestinal permeability (Fig. 1H) in *Mttp-IKO* mice. In line with the reduced Sirius staining, hepatic collagen content was reduced greater than 4-fold in *Mttp-IKO* mice (supplemental Fig. S1). Hepatic transcriptomic profiling was consistent with decreased inflammatory (F4/80 and Cx3cr1) and fibrogenic (Col1a1, Sma, and Desmin) signaling and increased DNL (Scd1, Scd2, and Fasn) in *Mttp-IKO* mice (Fig. 1I). These findings demonstrate that blocking chylomicron assembly mitigates hepatic steatosis and reduces both inflammatory and fibrogenic signaling following MCD feeding.

### Reduced oxidative stress, c-Jun N-terminal kinase, and nuclear factor kappa B activation in *Mttp-IKO* mice-fed MCD diet

**Fig. 2:**
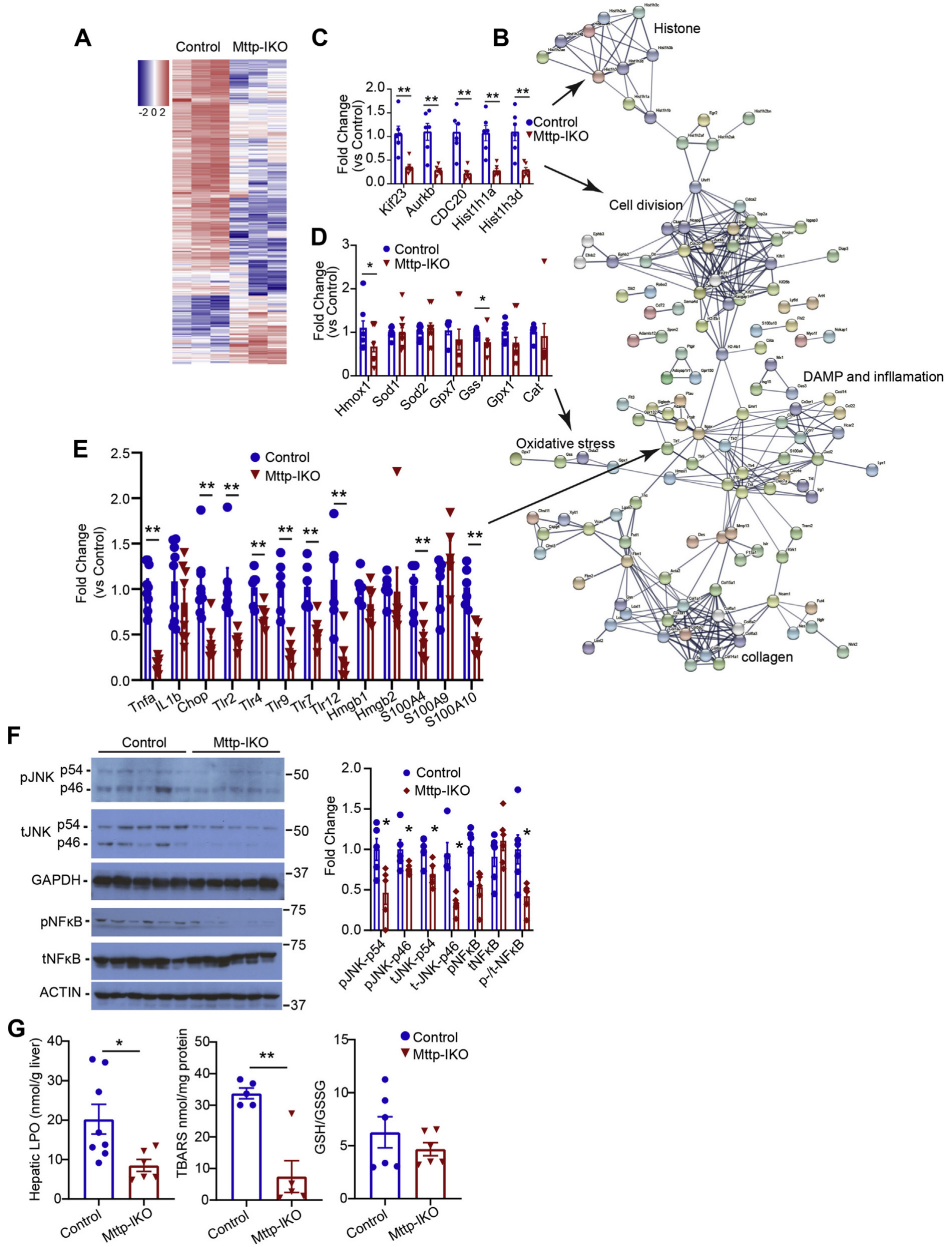
Intestinal *Mttp* deletion attenuates expression of liver damage-associated molecular patterns (DAMPs) and oxidative stress induced by MCD feeding in male mice. A: Heat map of 290 differentially expressed genes (log fold change [FC] ≥ ±2) in control and *Mttp-IKO* liver. B: STRING analysis of 205 differentially expressed genes with log (FC) ≤ −2 showing enriched pathways. C–E: Quantitative PCR validation of gene clusters involved in cell division (C), oxidative stress (D), and DAMP and inflammation (E) associated STRING pathways. F: Expression of total and phosphorylated JNK and NF-κB proteins in control and *Mttp-IKO* liver tissue. A representative Western blot (left) and quantitation of protein levels relative to controls after normalization to loading controls (GAPDH or ACTIN) are shown. G: Biochemical determination of hepatic lipid hydroperoxide (LPO, left panel) and TBARS (middle) levels. Right panel shows ratio of GSH to GSSG. For all bar graphs, data are presented as mean ± SEM, with n = 4–9/genotype. ∗*P* < 0.05 and ∗∗*P* < 0.01.

We next undertook transcriptomic profiling using RNA-Seq to understand in more detail the signaling pathways involved in mitigating inflammatory and fibrogenic injury in MCD-fed *Mttp-IKO* mice. Heat map and STRING analysis (Fig. 2A, B) revealed several groups of altered mRNAs including histone modifiers and cell cycle genes (Kif23, Aurkb, Hist1h1a, and Hist1h3d; Fig. 2C), oxidative stress modifiers (Hmox1 and Gss; Fig. 2D), and damage-associated molecular pathways/inflammatory signaling genes (Tnfa, Tlr4, Tlr7, Tlr12, S100A4, and S100A10; Fig. 2E). We further confirmed a role for altered inflammatory signaling through c-Jun N-terminal kinase (JNK) and nuclear factor kappa B (NF-κB)-related pathways, demonstrating reduced activation (decreased phospho-JNKp46, p54, and phosphorylated NF-κB; Fig. 2F). Similarly, we found reduced TBARS in MCD-fed *Mttp-IKO* mice, without a change in overall antioxidative capacity as evidenced by the glutathione/GSSG ratio (Fig. 2G). Those findings, coupled with the aforementioned observations, suggest that blocking chylomicron assembly in MCD-fed *Mttp-IKO* mice reduces hepatic steatosis and mitigates inflammatory and fibrogenic signaling through a combination of pathways, including altered cell signaling, damage-associated molecular pathway, and oxidative stress pathways.

### Conditional intestinal *Mttp* deletion reverses steatosis but not inflammation or fibrosis in MCD-fed mice

**Fig. 3:**
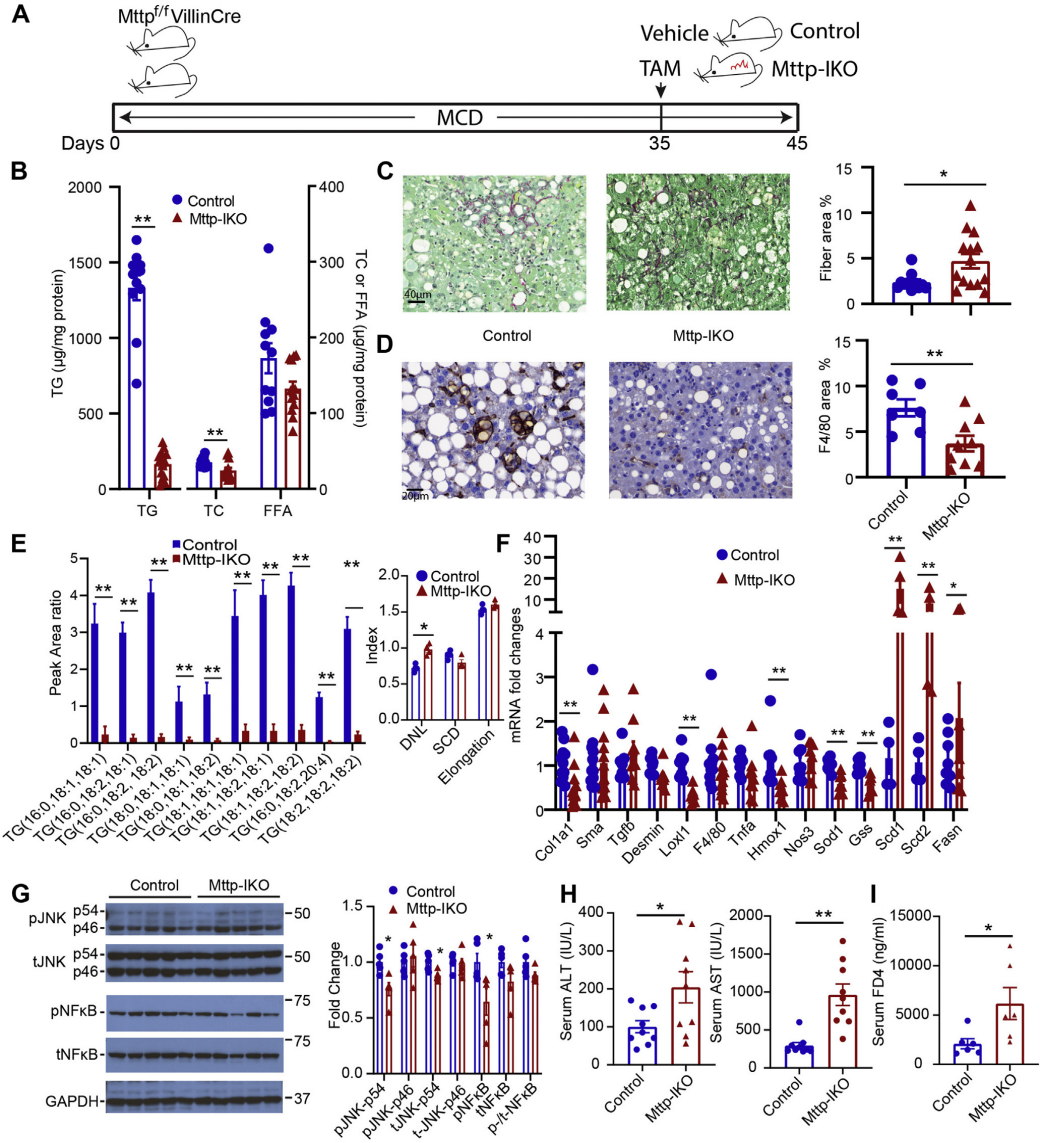
Short-term (10 days) intestinal *Mttp* deletion mitigates hepatic steatosis but not fibrosis. A: Schematic diagram of experimental design. B: Biochemical quantitation of hepatic lipid content (n = 11–13/genotype). C: Representative images of Sirius red-stained liver tissue (200×) and quantitation of fibrotic area (right panel, n = 11–13/genotype; both male and female mice). D: Representative images of hepatic F4/80 staining (400×) and quantitation of F4/80-stained area (right panel, n = 7–9/genotype; both male and female mice). E: Relative abundance of the most abundant TG species presented as peak area ratio, with DNL, SCD, and elongation indices calculated from all TG species shown on right (n = 4/genotype). F: mRNA expression of genes related to fibrosis, inflammation, and lipogenesis (n = 7–13/genotype). G: Expression of total and phosphorylated JNK and NF-κB proteins in control and *Mttp-IKO* liver tissue. Representative Western blot (left) and quantitation of relative protein levels are shown, with levels of GAPDH as a loading control. H: Serum ALT (left) and AST levels (n = 9/genotype). I: Serum FITC-dextran (FD4) levels 2 h after oral gavage (n = 6/genotype). For all panels, data are presented as mean ± SEM. ∗*P* < 0.05 and ∗∗*P* < 0.01.

Based on the aforementioned demonstration that blocking chylomicron assembly produced a significant effect on the development of hepatic steatosis and inflammatory-fibrogenic signaling, we asked if conditional intestinal *Mttp* deletion would also reverse those phenotypes, once established. We fed groups of *Mttp*^*f/f*^ Villin Cre ER^T2^ mice an MCD diet for 5 weeks to induce steatosis, inflammation, and fibrosis (Fig. 3A) and then treated mice with either TAM or vehicle and studied them after a further 10 days of MCD feeding. Even this short period of blocked chylomicron assembly produced >90% reduction in hepatic TG content (Fig. 3B), which was accompanied by an increase in both fibrosis (Fig. 3C) and hydroxyproline content (28 ± 2 → 69 ± 10 μg/mg protein; *P* < 0.01) despite decreased F4/80 staining (Fig. 3D). TG FA distribution showed a similar shift as noted previously in MCD-fed *Mttp-IKO* mice (compare Fig. 3E with Fig. 1C), along with increased DNL (Fig. 3E, F). Transcriptomic markers of fibrogenic (Col1a1, Sma, Tgfb, and Desmin) and inflammatory signaling genes (Tnfa and Loxl1) showed inconsistent changes with either a decrease or no change in MCD-fed *Mttp-IKO* mice (Fig. 3F), whereas markers of JNK and NF-κB signaling likewise were decreased or unchanged (Fig. 3G). In addition, markers of hepatic inflammation (ALT and aspartate aminotransferase) and intestinal permeability were increased in MCD-fed *Mttp-IKO* mice (Fig. 3H, I). Those findings suggest that short-term (10 days) induction of blocked chylomicron assembly in the setting of established steatosis and fibrosis produces substantial reductions in hepatic TG but no consistent improvement in inflammatory or fibrogenic signaling. The relatively brief time frame for this intervention raised the possibility that resolution of the inflammatory and fibrogenic signaling components of liver injury with MCD feeding might require an extended period following reduction of hepatic steatosis.

**Fig. 4:**
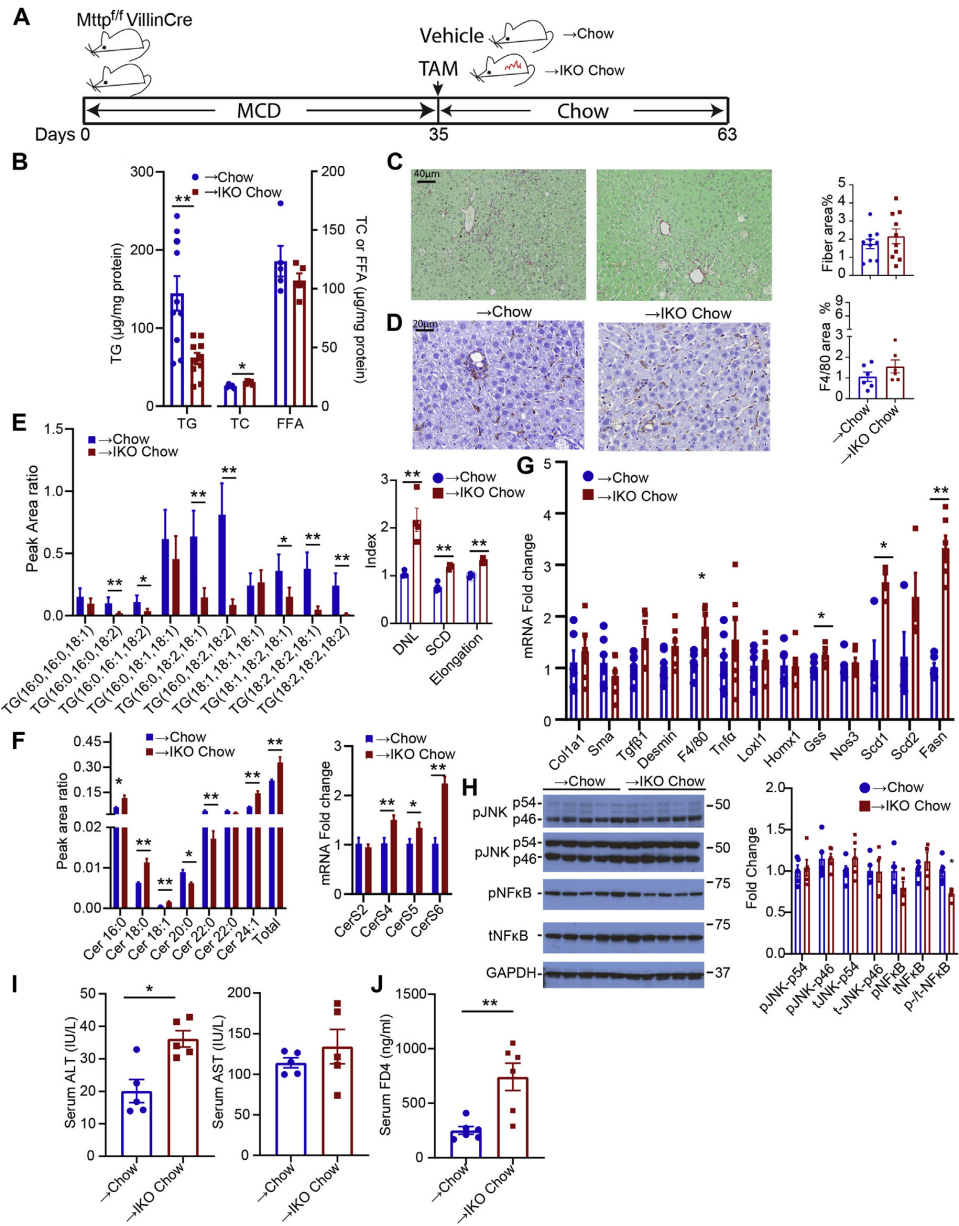
Switching from MCD to chow reverses steatosis but not inflammatory or fibrogenic signaling in male mice with intestinal *Mttp* deletion. A: Schematic diagram of experimental design. B: Biochemical quantitation of hepatic lipid content (n = 5–10/genotype). C: Representative images of Sirius red-stained liver tissue (200×) and quantitation of fibrotic area (right panel, n = 10/genotype). D: Representative images of hepatic F4/80 staining (400×) and quantitation of F4/80-stained area (right panel, n = 6/genotype). E: Relative abundance of the most abundant TG species presented as peak area ratio (n = 4/group). Right panel shows indices of lipogenesis (DNL), saturation, and elongation calculated from the relative abundance of all TG species. F: Relative abundance of hepatic ceramide (Cer) species, with total Cer peak area ratio shown on the far right. Right panel shows mRNA expression of Cer synthase isoforms in liver tissue. G: mRNA expression of genes related to fibrosis, inflammation, and lipogenesis (n = 6/genotype). H: Levels of total and phosphorylated JNK and NF-κB protein in →chow and →IKO chow liver tissue. A representative immunoblot and quantitation of relative expression are shown, with GAPDH presented as a loading control. n = 5/genotype (I) serum ALT (left) and AST levels. n = 5/genotype. J: Serum FITC-dextran FD4 levels 2 h after oral gavage. For all bar graphs, ∗*P* < 0.05 and ∗∗*P* < 0.01.

**Supplemental Fig. S2:**
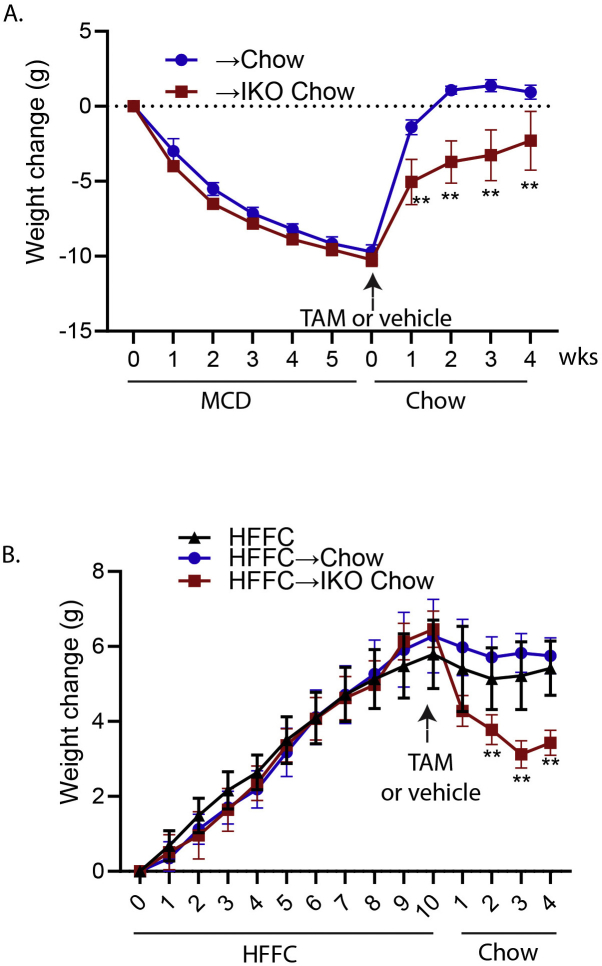


**Supplemental Fig. S3:**
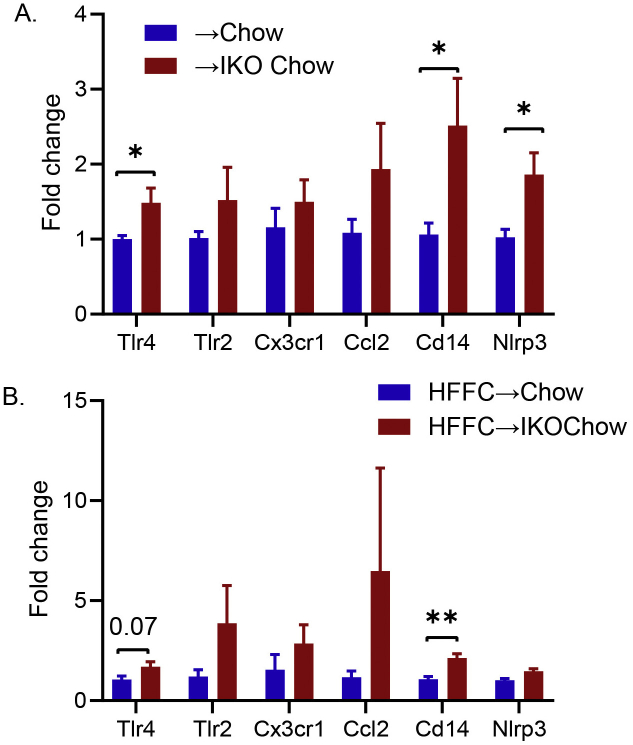


Based on those possibilities, we next asked if extending the time following induction of blocked chylomicron assembly would permit further resolution of the inflammatory/fibrogenic injury phenotype. Our initial studies extending the period following TAM administration with *Mttp-IKO* mice consuming an MCD diet revealed continued weight loss and increased lethality (data not shown). To circumvent this problem, we elected to feed groups of *Mttp*^*f/f*^ Villin Cre ER^T2^ mice an MCD diet for 5 weeks, then switched them to regular chow with either TAM or vehicle administration, and studied them after a further 4 weeks (Fig. 4A). Weight gain resumed in both groups upon switching to chow, although lower in *Mttp-IKO* versus control mice (supplemental Fig. S2A). Both groups demonstrated reduction in hepatic TG content after 4 weeks of chow feeding, but hepatic TG was again lower in *Mttp-IKO* versus control mice (Fig. 4B). Fibrosis, assessed by Sirius red staining and histopathologic review, was no different between groups (Fig. 4C) nor was there a difference in F4/80 staining (Fig. 4D). The TG FA species distribution again recapitulated findings from earlier studies (Figs. 1C, 3E), and DNL, SCD, and FA elongation indices were all increased in *Mttp-IKO* mice (Fig. 4E). In addition, we observed an increase in both total hepatic Cer content as well as in 16:0, 18:0, 18:1, and 24:1 species in *Mttp-IKO* mice, with a relative decrease in 20:0 and 22:0 species (Fig. 4F). Those changes were further extended with transcriptomic profiles showing increased mRNA abundance of CerS4, CerS5, CerS6 (Fig. 4F, right panel) as well as Scd1, Scd2, and Fasn in livers of *Mttp-IKO* mice (Fig. 4G). By contrast, other parameters of inflammatory (Tnfa and Loxl1) and fibrogenic (Col1a1, Sma, and Tgfb) signaling were no different by genotype (Fig. 4G) nor were there differences by genotype in JNK and NF-κB signaling (Fig. 4H). Those findings suggest that those signaling pathways identified previously in the preventive experiments (Figs. 1I, 2A–E) were not modified in the setting of conditionally impaired chylomicron assembly following established steatosis and fibrosis. As in the preventive experiments (Fig. 1G, H), serum ALT and FD-4 levels were increased in *Mttp-IKO* mice following reversal to chow, suggesting hepatocyte injury and impairment of intestinal barrier function (Fig. 4I, J), as well as upregulated expression of a subset of immune response genes (supplemental Fig. S3A).

### Conditional intestinal *Mttp* deletion reverses steatosis but not inflammation or fibrosis in HFFC-fed mice

**Fig. 5:**
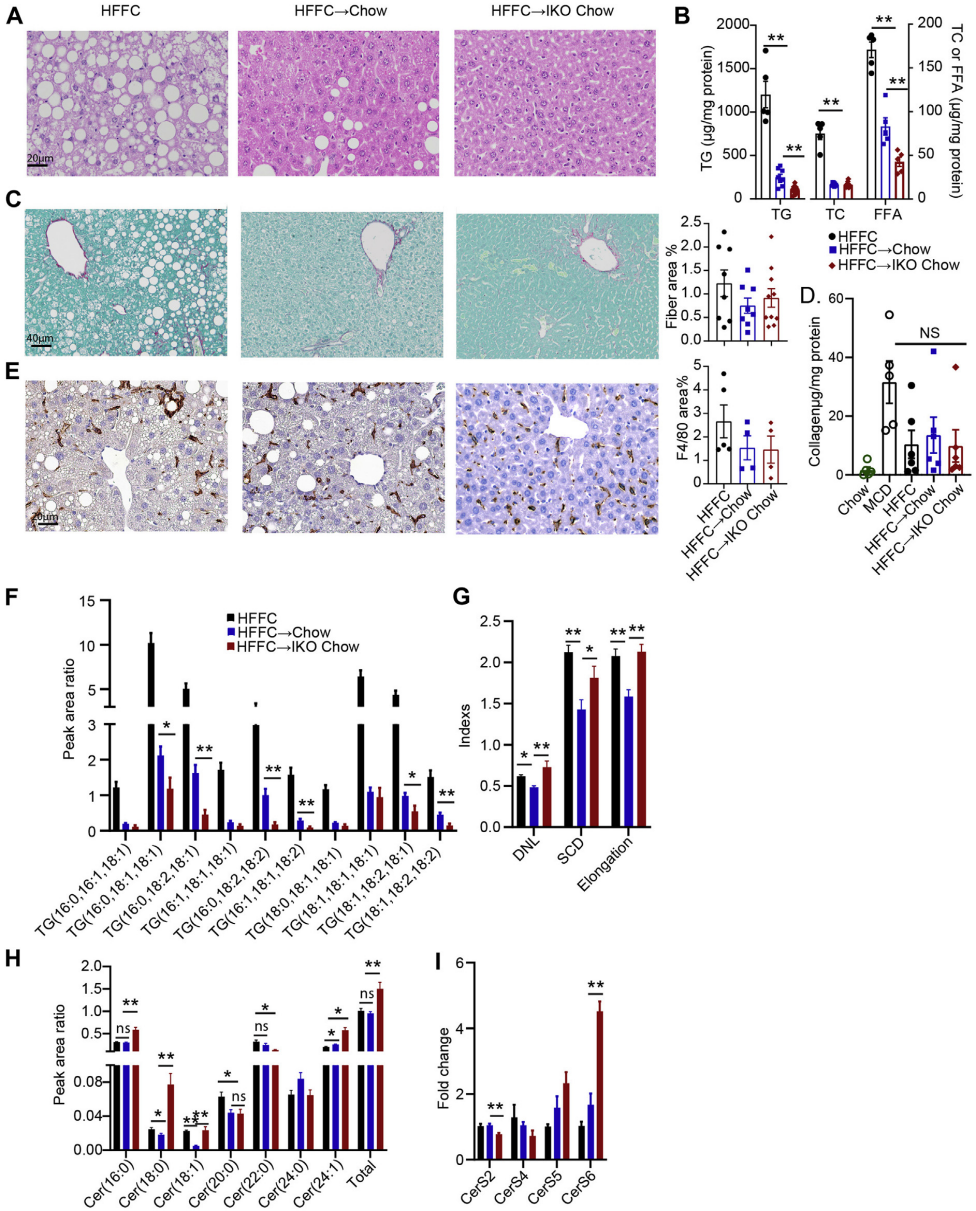
Switching from HFFC to chow diet reverses steatosis but not inflammatory or fibrogenic signaling in male *Mttp-IKO* mice. Abbreviations for all panels: HFFC, mice fed HFFC for 14 weeks; HFFC → chow, mice fed HFFC for 10 weeks and then chow for 4 weeks; HFFC → IKO chow, mice fed HFFC for 10 weeks and then chow for 4 weeks concomitant with induction of intestinal *Mttp* deletion. A: Representative images of H&E-stained liver tissue (400×). B: Biochemical quantitation of hepatic lipid content, n = 5–10/group. C: Representative images of Sirius red-stained liver tissue (200×), with quantitation of fibrotic area (right panel, n = 8–10/group). D: Collagen content determined by hydroxyproline detection and normalized to protein content, n = 4–5/group. E: Representative images of F4/80 staining (400×), with quantitation of F4/80-stained area shown at right (n = 4–5/group). F: Relative abundance of the most abundant TG species presented as peak area ratio (n = 5/group). G: Indices of DNL, saturation (SCD), and elongation calculated from peak area of all TG species (n = 5/group). H: Relative abundance of hepatic ceramide (Cer) species, with total Cer peak area ratio shown on the far right (n = 5/group). I: mRNA expression of hepatic Cer synthase isoforms (n = 5/group). For all panels, ∗*P*< 0.05 and ∗∗*P* < 0.01.

We next turned to an alternative dietary model of NAFLD/NASH with inflammation and fibrosis, using a HFFC model, which has been utilized in various subtle iterations by several investigators (19, 20, 28, 29, 30). After 10 weeks of feeding, groups of *Mttp*^*f/f*^ Villin Cre ER^T2^ mice were either maintained on HFFC as control or transitioned to a regular chow diet with (+TAM) or without (+vehicle) induction of intestinal *Mttp* deletion for a further 4 weeks. As shown in Fig. 5A, 14 weeks of HFFC feeding induced gross macrovesicular steatosis and periportal and pericentral fibrosis (Fig. 5C), along with diffuse F4/80 staining (Fig. 5E). Upon switching to a chow diet, we observed weight loss in *Mttp-IKO* mice (supplemental Fig. S2B). Groups of *Mttp*^*f/f*^ Villin Cre ER^T2^ (vehicle injected and control) and *Mttp-IKO* mice transitioned to a chow diet for 4 weeks showed a striking reduction in hepatic steatosis (Fig. 5A), with a greater reduction in *Mttp-IKO* mice (1,200 ± 153 → 294 ± 34 control vs. 102 ±15 μg/mg protein in *Mttp-IKO*), and a concomitant decrease in hepatic content of TG, cholesterol, and FFA (Fig. 5B). However, despite the reversal of hepatic steatosis in both groups, there were no significant changes in fibrosis—as assessed by either Sirius staining (Fig. 5C) or hepatic hydroxyproline content (Fig. 5D). Likewise, there was no significant change in F4/80 staining in either of the groups switched to chow (Fig. 5E). There were differential shifts in TG FA species in *Mttp*^*f/f*^ Villin Cre ER^T2^ (vehicle injected and control) and *Mttp-IKO* mice transitioned to a chow diet (Fig. 5F), predominantly involving polyunsaturated FA species. In addition, and similar to the adaptations observed previously in MCD-fed mice, indices of DNL, SCD, and FA elongation were all increased in chow-*Mttp-IKO* mice (Fig. 5G). Total hepatic Cer content was also higher in HFFC-fed mice transitioned to chow with induction of *Mttp-IKO*, with selective increases in C16:0, C18:0, and C24:1 ceramides in chow-*Mttp-IKO* mice (Fig. 5H). In line with the changes in hepatic Cer content, mRNA expression of hepatic CerS6 was increased in chow-fed *Mttp-IKO* mice (Fig. 5I).

**Fig. 6:**
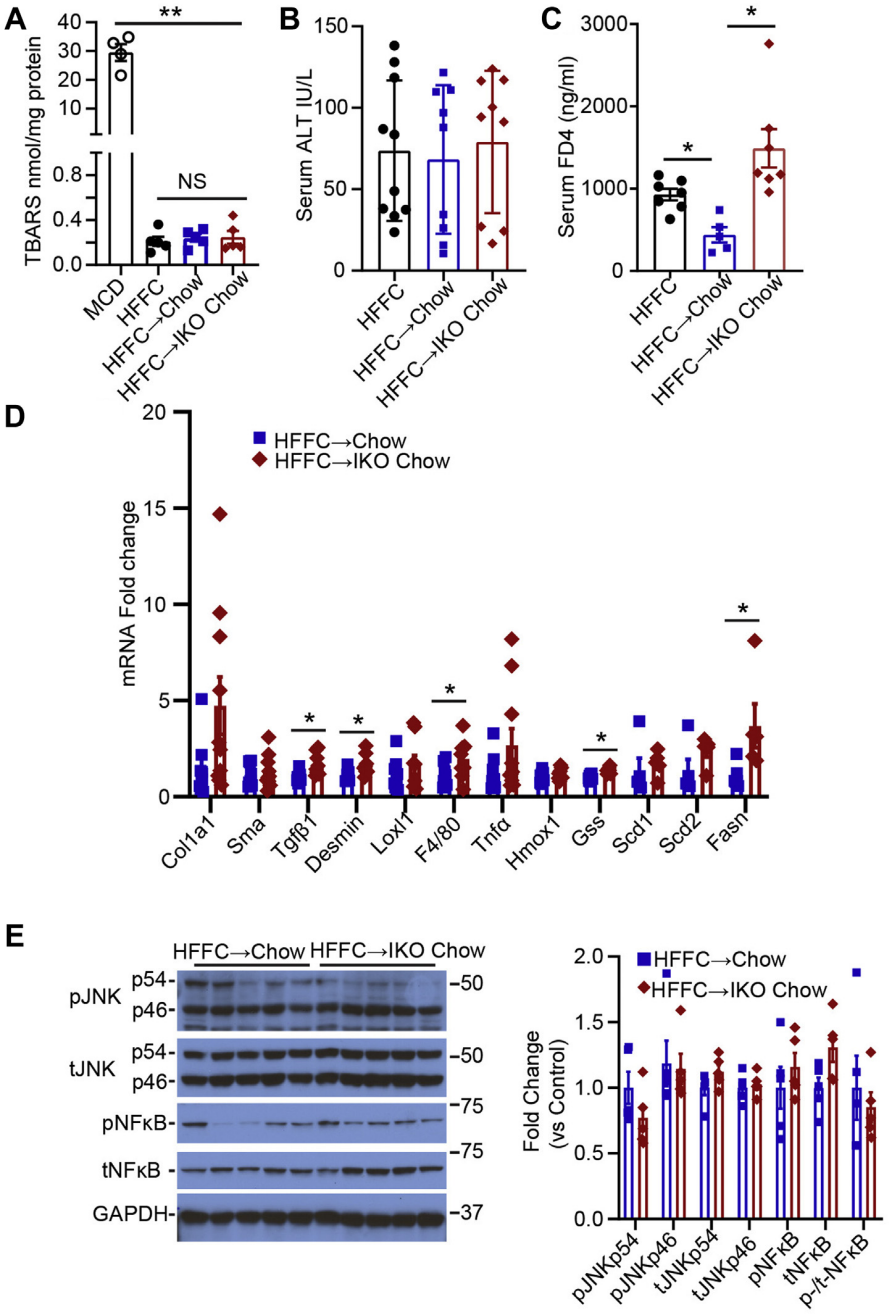
Levels of oxidative stress and expression of NASH-related genes are unchanged in male *Mttp-IKO* mice following switch from HFFC to chow diet. A: Liver TBARS content, n = 4–5/group, with levels in MCD-fed control mice shown for comparison. B: Serum ALT, n = 9–10/group. C: Serum FITC-dextran FD4 levels 2 h after oral gavage, n = 5–7/group. D: mRNA expression of genes related to fibrosis, inflammation, and lipogenesis (n = 6/genotype). E: Expression of total and phosphorylated JNK and NF-κB proteins. A representative immunoblot and quantitation of relative protein expression levels are shown (n = 5/group). Abbreviations are as outlined in the legend to Fig. 5. For all panels, ∗*P* < 0.05 and ∗∗*P* < 0.01.

**Fig. 7:**
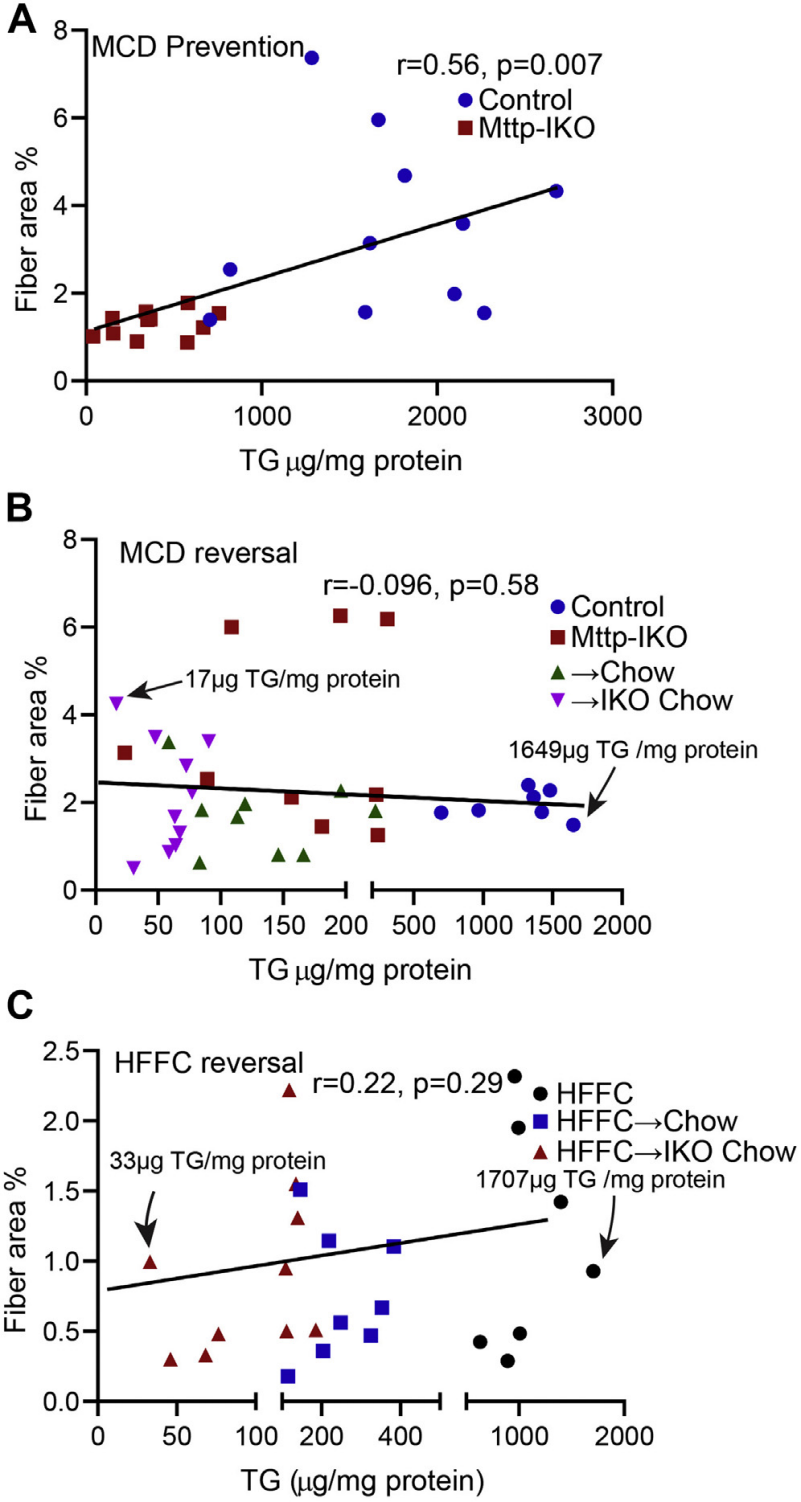
Correlation between hepatic TG content and fibrotic area. A: A positive association between hepatic TG content and fibrotic area in male *Mttp-IKO* mice-fed MCD diet for 3 weeks. B: No correlation between hepatic TG content and fibrotic area in the MCD reversal experiments, including data from male and female mice ± *Mttp* deletion with continued MCD feeding (control, *Mttp-IKO*) and mice ± *Mttp* deletion concomitant with switch to chow diet (→chow, →IKO chow). C: No correlation between hepatic TG content and fibrotic area in the HFFC reversal experiments. Groups include male mice-fed HFFC for 14 weeks, mice fed HFFC for 10 weeks, chow for 4 weeks (HFFC → chow), and mice fed HFFC for 10 weeks, chow for 4 weeks with intestinal *Mttp* deletion (HFFC → IKO chow). Pearson correlation coefficient (Pearson's r) and *P* values are shown on each graph.

We found no changes in hepatic TBARS in any of the groups fed HFFC diet (Fig. 6A), in contrast to the findings in MCD-fed mice. Similarly, there was no change in serum ALT across the HFFC-fed groups (Fig. 6B). Intestinal permeability was increased in *Mttp*^*f/f*^ Villin Cre ER^T2^ mice fed HFFC, which was reversed upon transition to chow (Fig. 6C), whereas chow-fed *Mttp-IKO* mice exhibited a continued increase in intestinal permeability (Fig. 6C). Hepatic transcriptomic profiling revealed a nonsignificant trend to increased Col1a1 and increased Tgfb, and Desmin mRNA abundance in chow-fed *Mttp-IKO* mice, along with increased F4/80 mRNA (Fig. 6D). We also observed alterations in a subset of hepatic immune response gene in chow-fed *Mttp-IKO* mice (supplemental Fig. S3B). Consistent with the increased DNL index, *Mttp-IKO* mice also exhibited increased Fasn expression (Fig. 6D). Parameters of JNK and NF-κB activation were not significantly different by genotype in either group transitioned to chow diet (Fig. 6E). These findings, considered together with the histologic and aforementioned lipidomic data, suggest two major conclusions. First, the inflammatory signaling and fibrosis accompanying HFFC feeding is less severe than observed with MCD feeding, and second, despite almost complete resolution of hepatic steatosis, there is ongoing inflammatory and fibrogenic signaling that perpetuates the underlying injury phenotypes. We found that hepatic steatosis, reflected by hepatic TG content, was positively correlated (as expected) with development of fibrosis in animals fed an MCD diet (Fig. 7A). However, over a >50–90-fold range of hepatic TG, there was no correlation between hepatic steatosis and fibrosis following the reversal interventions in either MCD-fed or HFFC-fed mice (Fig. 7B, C).

## Discussion

The major conclusions of our studies are that blocking chylomicron assembly through conditional deletion of intestinal *Mttp* mitigates the development of hepatic steatosis in MCD-fed mice and reduces inflammatory and fibrogenic signaling. However, when applied in either of two models of established steatosis and fibrosis, blocking chylomicron assembly resulted in efficient lipid mobilization and reduced hepatic steatosis, yet failed to reverse inflammatory signaling and fibrosis. Several features of the approach and conclusions of this work merit further discussion in the context of altered gut-liver signaling in the pathogenesis and therapeutic targeting of NAFLD.

Multiple lines of evidence point to a role for impaired intestinal barrier function and bacterial translocation in the pathogenesis of NAFLD and NASH (31). Our prior findings in *Mttp-IKO* mice demonstrated increased circulating levels of TNFα and exaggerated colonic inflammation and injury during experimental colitis (17). Those findings raised the suspicion that if *Mttp-IKO* mice were fed an MCD diet, a model known to promote intestinal barrier disruption and systemic inflammation (32), there would be increased liver injury. However, that was not the case. Despite the further impairment of barrier function in *Mttp-IKO* mice as evidenced by elevated serum FD-4 levels, *Mttp-IKO* mice exhibited reduced inflammatory and fibrogenic signaling. Those findings encouraged us to consider whether conditional impairment of chylomicron assembly might also reverse some of the key features in models of established NAFLD/NASH. We were further encouraged by other work showing that mice with intestine-specific deletion of high mobility group box 1 develop impaired intestinal lipid absorption with decreased chylomicron formation, yet were protected against diet-induced NASH (33).

However, despite the protection offered to *Mttp-IKO* mice when fed an MCD diet, the induction of intestinal *Mttp* deletion following the development of established fibrosis not only failed to promote resolution but also rather led to exaggerated injury and sustained inflammatory signaling. We modified our approach in the reversal experiments with MCD feeding because of continued weight loss and mortality associated with prolonged exposure to this diet in *Mttp-IKO* mice, but even 10 days of impaired chylomicron assembly in mice fed an MCD diet resulted in worse fibrosis. Extending the reversal time to 28 days, along with a switch to regular chow, also failed to improve fibrosis in *Mttp-IKO* mice. Because of concerns with MCD feeding as a representative model of human NASH (34), we turned to a second model in which high-fat feeding is supplemented by fructose and cholesterol to augment injury and inflammatory signaling (19, 20, 29, 30). This model, herein referred to as HFFC and widely used in various subtle iterations, produces less fibrotic injury than MCD feeding but recapitulates other elements of human NAFLD/NASH, including obesity, hepatic steatosis, and metabolic dysfunction with insulin resistance (34). We observed mild fibrosis in mice fed an HFFC diet for 14 weeks but, as noted in MCD-fed mice, we observed no fibrosis reversal in *Mttp-IKO* mice that were switched to a chow diet for 4 weeks, despite virtually complete elimination of hepatic steatosis.

It is worth noting that the decreased overall abundance of hepatic lipid in MCD-fed *Mttp-IKO* mice was accompanied by shifts in FA species distribution, with relative preservation of 16:1 and 18:1 species accompanied by upregulation of Scd1 mRNA expression and indices of DNL. Those findings suggest that a component of the adaptation to blocked chylomicron assembly in *Mttp-IKO* mice involves increased hepatic lipogenesis, which was observed in prior studies in chow-fed *Mttp-IKO* mice (11, 12), along with increased expression of hepatic Scd1 (12). Earlier work showed that germ line *Scd1* null mice were protected against hepatic steatosis when fed an MCD diet, yet exhibited more severe injury (35), whereas other work showed that *Scd1* null mice were protected against both hepatic steatosis and inflammation in the setting of alcohol-induced injury (36). Those findings, coupled with the current observations in *Mttp-IKO* mice, suggest that the adaptive induction of Scd1 expression and function may mitigate the reversal of inflammatory signaling in the setting of established steatosis and fibrotic injury and further suggest that the overall distribution of FA species rather than simply the quantity of hepatic lipid is a key component of the injury phenotypes observed. Those suggestions align with other work demonstrating that switching WT mice from an HFFC diet to chow for 8 weeks reversed the hepatic steatosis but failed to normalize hepatic FA distribution among neutral and polar lipids (28). Among the key lipid signaling molecules, we found increased hepatic Cer FA abundance in *Mttp-IKO* mice in both dietary reversal models. Previous studies demonstrated increased hepatic Cer content following MCD feeding in mice (37, 38), and other work has demonstrated that accumulation of specific Cer species (C16:0 and C24:1) correlated with fibrogenic activation in vitro in hepatic stellate cells (39). It is possible that altered immune signaling in the livers of mice plays a role in the continued inflammatory and fibrogenic signaling following transition from either MCD and/or HFFC feeding, based on increased expression of damage-associated molecular patterns and mediators, including Tlr4, Cd14, and Nlrp3 (supplemental Fig. S3), but further studies will be required to consolidate those findings.

Previous findings with regard to the metabolic signaling pathways associated with impaired chylomicron assembly in *Mttp-IKO* mice suggest similarities in the adaptive pathways to those observed in rodents and humans undergoing bariatric surgery interventions such as Roux-en-Y gastric bypass (14). These include alterations in enterohepatic bile acid signaling mediated via farnesoid X receptor and its downstream targets fibroblast growth factor 15 as well as through TGR5, which produce favorable metabolic shifts in glucose and lipid metabolism and amelioration of hepatic steatosis (40, 41). However, while bariatric surgical interventions have been reported to successfully reverse the metabolic complications of extreme obesity in patients, there are inconsistent effects on the reversal of established hepatic fibrosis (7, 42, 43, 44). Our studies were not designed to evaluate the role of weight loss, but we note that both genotypes fed the MCD diet exhibited weight loss during active feeding and resumed weight gain upon switching to a chow diet, with or without induction of intestinal *Mttp* deletion (supplemental Fig. S2A). Mice fed the HFFC diet for 10 weeks were obese, and there was weight loss in mice switched to regular chow along with induction of intestinal *Mttp* deletion (supplemental Fig. S2B). Nevertheless, neither group of mice exhibited reduction in fibrosis in the context of those interventions. In addition, fasting glucose levels were unchanged at terminal sacrifice in HFFC-fed mice transitioned to chow but were reduced (as expected) in HFFC-fed mice transitioned to *Mttp-IKO* plus chow (supplemental Fig. S4). Those findings are consistent with our prior demonstration that insulin signaling is enhanced in *Mttp-IKO* mice and make it unlikely that insulin resistance contributes to the continued inflammatory and fibrogenic signaling observed. Other recent findings demonstrated that *Mttp-IKO* mice exhibit increased circulating levels of incretins including GLP1, in association with delayed gastric emptying and reduced hepatic gluconeogenesis (14). However, a recent phase II trial of the GLP1 agonist semaglutide demonstrated weight loss and significant resolution of NASH (vs. placebo) but no significant decrease in hepatic fibrosis (8). Taken together with our current findings, these observations raise implications in understanding the potential for dietary and pharmacologic interventions in patients with NAFLD. For example, we speculate that the beneficial effects of sustained weight loss on reversal of fibrosis may require a much longer period following intervention, compared with the effects of even modest weight loss on hepatic steatosis. A caveat in this suggestion is that prolonged impairment of chylomicron assembly with defective fat absorption may be associated with essential fatty acid deficiency. We did not specifically examine this possibility, but future studies might incorporate determinations of serum Mead acid (20:3; n-9) and other markers of essential fatty acid deficiency as previously proposed from our own and other laboratories (45, 46).

Earlier findings in mice treated with antisense oligonucleotides to diacylglycerol acyltransferase 2 offer further insights in attempting to understand the disconnect between resolution of hepatic steatosis and inflammatory/fibrogenic signaling in experimental NASH (47). Those workers treated groups of mice following MCD feeding and observed that the antisense-treated mice exhibited reduced steatosis, yet worse inflammation and fibrosis than controls (47). Our findings build upon those observations by providing an independent approach to modifying hepatic lipid content (i.e., blocking chylomicron assembly) that occur despite adaptive changes in hepatic lipogenesis.

We chose to examine the therapeutic potential for conditionally blocking chylomicron assembly as an intervention to mitigate progression and promote reversal of NAFLD because of the continued unmet need to understand the factors associated with liver-related morbidity and mortality associated with advanced forms of the disease including NASH (1). Our focus on surrogate end points as intervention targets is highlighted by recent studies from several population cohorts demonstrating that hepatic fibrosis (as confirmed by liver biopsy) is a major predictor of both morbidity and mortality in patients with NAFLD and NASH, even when corrected for confounding factors (48). Our findings suggest that modifying chylomicron assembly through approaches that impair intestinal *Mttp* expression or function may represent a viable therapeutic option for reversing established steatosis. That being said, additional or alternative strategies will be required to mitigate inflammatory and fibrogenic signaling in patients with NASH.

## Data availability

All data are contained in the body of the article. RNA-Seq data files are deposited in a publicly accessible repository (GSE182130) available following acceptance.

## Supplemental data

This article contains supplemental data.

## Conflict of interest

The authors declare that they have no conflicts of interest with the contents of this article.

## Figures and Tables

**Supplemental Fig. S4:**