# Supplementary material for: Inhibition of chylomicron assembly leads to dissociation of hepatic steatosis from inflammation and fibrosis
Source: J Lipid Res. 2021 Sep 24;62:100123. doi: 10.1016/j.jlr.2021.100123 (PMC8515302; doi:10.1016/j.jlr.2021.100123)
Supplement: Supplemental Table S1 [file mmc1.docx]

Supplemental table 1 oligodeoxyribonucleotide primer sequences for real-time quantitative PCR

| Gene | Forward | Reverse |
| --- | --- | --- |
| Aurkb | ACCAGCTTCGCCGAGAGA | GTTGAAGGATGTTGGGATGTTTC |
| Cat | GGCTTTTGACCCAAGCAATATG | CGGCCCTGAAGCATTTTGT |
| Cdc20 | CAACGCAGTGCTTCTCAAATG | CGGCTGGTTTTCCTTGCTT |
| CerS2 | ATGCTCCAGACCTTGTATGACT | CTGAGGCTTTGGCATAGACAC |
| CerS4 | CACGCTTCGGCAGACTCAA | GGATAAGGAAGGCCGATCCT |
| CerS5 | AGGATGACCGCAGTGATGTAGA | TGTTTTTGTGGGTTGTCTCATCTT |
| CerS6 | TCAACAACATGGCCCGAGTA | TTGGCGTAATTTGCCATTTTG |
| Col1a1 | CACGGCTGTGTGCGATGA | TCGCCCTCCCGTCTTTG |
| Chop | CCACCACACCTGAAAGCAGAA | TGAAAGGCAGGGACTCA |
| CX3cr1 | CGGTCTGGTGGGAAATCTGT | TTGGGCTTCCGGCTGTT |
| Desmin | GGAAATCCAACTAAGAGAAGAAGCA | CTAGAGTGGCTGCATCCACATC |
| Fasn | TCCTGGAACGAGAACACGATCT | GAGACGTGTCACTCCTGGACTTG |
| F4/80 | AAACACTTGGTGGTGTGAATGAGT | GCCTCGTTTACAGGTGCAGTAA |
| Gpx1 | CCTTGCCAACACCCAGTGA | CCGGAGACCAAATGATGTACTTG |
| Gpx7 | GGCCAACAGGAACCAGACA | TGCGGCGGGCAAAGT |
| Hist1h1a | AGCCCGCGGGACCTT | CTGCGCTCTTTGGAAGAAGAA |
| Hist1h3d | CCAAGCGTGTCACCATCATG | GGTCCAAATTCACTCACTACCCTAA |
| Hmox1 | CAGCCCCACCAAGTTCAAA | TCAGGTGTCATCTCCAGAGTGTTC |
| Hmgb1 | AAAGAAACTAGGAGAGATGTGGAACAA | AGCTTGGCAGCTTTCTTCTCA |
| Hmgb2 | CCGCGAGGAGCACAAGAA | CTTGGAGAACTCGGCGAAGT |
| IL1β | GGTGTGTGACGTTCCCATTAGAC | CCGACAGCACGAGGCTTTT |
| Kif23 | GGAGCTTGAGAGCCAGAATCA | GCTTCTAATCTGCGCTTGTCAGA |
| Lox | CAAGCCGCCCTCGGTACT | CGTCCATGCTGTGGTAATGTTG |
| Loxl1 | CCGCAGCAGTTCCCCTATC | CGCGGGATCGTAGTTCTCAT |
| Mmp2 | TGGGACAAGAACCAGATCACATA | AAAGCATCATCCACGGTTTCA |
| Mmp9 | GGGCCGCCCAGATGA | CAGGGAGAGCTGCTTCTGAAG |
| Mmp12 | GATGTGAGGCAGGAGCTCATG | GGCTTGATTCCTGGGAAGTG |
| Nos3 | TTGTCTGCGGCGATGTCA | GAATTCTCTGCACGGTTTGCA |
| Scd1 | TCGAAGGACCCGAGGTGTT | CACCTCTTAGCAGCTACTTACAGACACT |
| Scd2 | GTACCGCTGGCACATCAACTT | ACACTCTCTTCCGGTCGTAAGC |
| SMA | CCAGAGCAAGAGAGGGATCCT | TGTCGTCCCAGTTGGTGATG |
| Sod1 | CCCGGCGGATGAAGAGA | ATTGGCCACACCGTCCTTT |
| Sod2 | TTAACGCGCAGATCATGCA | GGTGGCGTTGAGATTGTTCA |
| S100A4 | GGAGGAGGCCCTGGATGTA | CACCCTCTTTGCCTGAGTATTTG |
| S100A9 | TCATGGAGGACCTGGACACA | CAGCATCATACACTCCTCAAAGCT |
| S100A10 | CTGGACCAGTGCCGAGATG | AGCCCTGCCACTAGTGATAGAAA |
| Tgfβ1 | TGCCCGAACCCCCATT | TTGCTCCACACTTGATTTTAATCTCT |
| Tlr2 | CACCACTGCCCGTAGATGAA | GCCTCGGAATGCCAGCTT |
| Tlr4 | GCAGCAGGTGGAATTGTATCG | TGTGCCTCCCCAGAGGATT |
| Tlr7 | CAGTGAACTCTGGCCGTTGA | CAAGCCGGTTGTTGGAGAA |
| Tlr9 | GCAGCTGAACCTCAAGTGGAA | TGGTCATGTGGCAAGAGAAGTG |
| Tlr12 | CTGCCACTGTGCCAATGC | ATATGTACGTTTTAGTGGACCGCTTA |
| Tnfα | GGTCCCCAAAGGGATGAGAA | TGAGGGTCTGGGCCATAGAA |
