# Supplementary material for: Inhibition of chylomicron assembly leads to dissociation of hepatic steatosis from inflammation and fibrosis
Source: J Lipid Res. 2021 Sep 24;62:100123. doi: 10.1016/j.jlr.2021.100123 (PMC8515302; doi:10.1016/j.jlr.2021.100123)
Supplement: Supplemental Figure Legend [file mmc2.docx]

**Supplemental Figure S1.** Liver collagen content was determined by hydrolysis and hydroxyproline detection, normalize to its protein content. n=5 /group. **p<0.01.

**Supplemental Figure S2.** Body weight was recorded once a week. Weight change against starting weight was plot against weeks on the different diet and experimental condition as indicated in x-axis. (A) After fed MCD for 5 weeks, mice were switched to chow diet and injected either by TAM (→IKO Chow) or vehicle (→Chow). n=8-10/ group (B) After fed TFFC for 10 weeks, mice were kept on HFFC (in black) or switched to chow diet plus injected either with TAM (HFFC→IKO Chow, in red) or vehicle (HFFC→Chow) in blue). n=8-10 /group. *p<0.05, **p<0.01.

**Supplemental Figure S3.** Hepatic mRNA expression of immune response genes related to damage (and /or microbial) associated molecular patterns was quantitated by qPCR. (A) *Mttp^f/f^* Villin Cre ER^T2^ mice were fed MCD for 5 weeks, then switched to chow diet with groups of mice injected with TAM (→IKO Chow) or vehicle (→Chow) and sacrificed 35 days later. n=6/genotype. (B). *Mttp^f/f^* Villin Cre ER^T2^ mice were fed HFFC for 10 weeks, then switched to chow diet with groups of mice injected with TAM (HFFC→IKO Chow ) or vehicle (HFFC→Chow) and sacrificed 4 weeks later. n=6-8/genotype. Data are presented as mean ± SEM, with * indicating p<0.05, ** indicating p<0.01

**Supplemental Figure S4.** Fasting serum glucose levels at sacrifice in the indicated genotypes following 14 weeks HFFC feeding (HFFC), versus 10 weeks HFFC+4 weeks chow (HFFC→Chow), versus 10weeks HFFC+ induction of *Mttp-IKO* with 4weeks chow (HFFC→IKO Chow). n=6/genotype. *p<0.05. ns: no significant difference.
